# Social Capital Changes After COVID-19 Lockdown Among Youths in China: COVID-19 Impact on Lifestyle Change Survey (COINLICS)

**DOI:** 10.3389/fpubh.2021.697068

**Published:** 2021-08-16

**Authors:** Bin Yu, Miyang Luo, Meijing Liu, Junmin Zhou, Shujuan Yang, Peng Jia

**Affiliations:** ^1^West China Second University Hospital of Sichuan University and Key Laboratory of Birth Defects and Related Diseases of Women and Children (Sichuan University), Ministry of Education, Chengdu, China; ^2^Xiangya School of Public Health, Central South University, Changsha, China; ^3^International Institute of Spatial Lifecourse Epidemiology (ISLE), Hong Kong, China; ^4^West China School of Public Health and West China Fourth Hospital, Sichuan University, Chengdu, China; ^5^Institute for Disaster Management and Reconstruction, Sichuan University, Chengdu, China

**Keywords:** COVID-19, social capital, mental health, youths, lockdown

## Abstract

**Introduction:** Social capital, the effective functioning of social groups through networks of relationships, can affect mental health and may be affected by COVID-19. We aimed to examine the changes in social capital before and after the COVID-19 lockdown among the Chinese youth.

**Methods:** A national convenience sample of 10,540 high school, undergraduate, and graduate students, from the COVID-19 Impact on Lifestyle Change Survey (COINLICS), reported their demographic and social capital information before and after the COVID-19 lockdown. Social capital was retrospectively measured at four levels: individual (ISC), family (FSC), community (CSC), and society (SSC). The changes of social capital were also compared across three educational levels.

**Results:** Overall, ISC and CSC scores generally decreased after lockdown (15.1 to 14.8 and 13.4 to 13.1, respectively), while FSC and SSC scores increased significantly (12.7 to 13.0 and 7.1 to 7.2, respectively). At the individual level, most participants showed a constant perceived social capital; more of the remaining participants showed decreased than increased ISC (30.5% vs. 17.0%) and CSC scores (28.4% vs. 19.1%), while more participants showed increased than decreased FSC (21.7% vs. 9.2%) and SSC scores (10.3% vs. 3.9%). Heterogeneities in social capital changes existed across educational levels.

**Conclusions:** Our findings would provide health professionals and policy-makers solid evidence on the changes in social capital of youths after lockdowns, and therefore help the design of future interventions to rebuild or improve their social capital after epidemics/disasters.

## Introduction

Social capital is broadly defined as the sum of trustworthy, reciprocal and resource-rich network connections (1). As a sophisticated formulation of the broader concepts of “social cohesion,” “social support,” “social integration,” or “civil society,” social capital is of great significance to both individuals and society (1, 2). From an individual perspective, social capital has been revealed as a crucial determinant of multiple health outcomes (e.g., adolescent well-being, mental health), with plausible pathways from social capital to health (1). From a society perspective, social capital is also proved as an asset to empower and mobilize a society and its members (3, 4). Especially highlighted is the crucial role of social capital when a nation's people face disasters or catastrophes (5). For instance, a survey in Japan showed that social capital buffered the effects of natural disasters and helped to resume groups' health during the 2011 Great East Japan Earthquake and Tsunami (5, 6). Given its crucial roles, preventing the possibility of dramatic post-disaster decline in social capital deserves attention, especially for youths who are fairly dependent on society and family (7). The youth might be more likely to show a significant change in social capital when facing disasters, which may directly or indirectly affect their mental health and also vary by age and level of maturity (i.e., youths of different levels of maturity may perceive social capital differently) (8, 9).

The coronavirus disease 2019 (COVID-19) that broke out nearly all over the world is undoubtedly disastrous (10). In China, to curb the spread of the epidemic, the government adopted strict policies including conducting a lockdown (11, 12). Thus, many factors closely related to social capital of the youth, such as social participation and interpersonal communication, may have undergone significant changes. For instance, even when social media platforms were available, face-to-face communication could not be achieved with the long period of social distancing and stay-at-home recommendations during the lockdown. Currently, despite the lockdown has been lifted, the abovementioned adverse situations have aroused the concern that social capital of youths might have been affected and changed. These changes might be negative because some factors, such as excessive reaction to the lockdown policy and poor psychological status (for stressful life events, extended home confinement, brutal grief information pollution on social media), might affect interpersonal or social cohesion (13–15). On the other hand, with effective emergency management, the whole society may have greater solidarity when facing disasters, leading to a positive change in social capital. However, the impacts of COVID-19 on social capital of the youth remain unknown in China. Furthermore, considering the heterogeneity in maturity and lifestyles (e.g., living at school or home) among youths, the level of social capital at baseline (i.e., at normal times before COVID-19 lockdown) and the degree of change in social capital after COVID-19 lockdown that may vary across educational levels, the social capital changes across educational levels are also examined.

This study aimed to examine differences in social capital in the months before COVID-19 lockdown was implemented (January 2020, also referred to as before lockdown) and after COVID-19 lockdown was lifted (May 2020, also referred to as after lockdown), as well as the variation in social capital changes across the educational levels, on the basis of a national convenience sample of 10,540 Chinese youths. Our findings would provide empirical evidence and references for targeted interventions of social capital reconstruction among youths in China, and may also benefit other countries which have encountered lockdown measures to different extents.

## Methods

### Data

The data used in this study were from the COVID-19 Impact on Lifestyle Change Survey (COINLICS), a national retrospective online survey designed by an expert panel consisting of epidemiologists, statisticians, health psychologists, and sociologists. A snowball sampling strategy was adopted to distribute the online questionnaire via social media platforms in May 2020 among youths at three educational stages (i.e., high school, college, and graduate students) in China (16). A total of 10,540 individuals completed the questionnaire anonymously. All subjects voluntarily participated in our study with informed consent, and the study was carried out in accordance with the Helsinki Declaration of 1964.

### Measurement of Social Capital

The individual social capital (ISC), family social capital (FSC), and community social capital (CSC) comprehensively reflect one's perception of social capital from peers/friends, family members, and neighbors, which have been proved to be associated with youths' health promotion or risk behaviors (17, 18). Also, measures taken by the government and relevant sectors to contain the COVID-19 pandemic have unprecedentedly attracted substantial social attention and possibly raised public trust, which could be reflected by the society social capital (SSC) (19).

The measurements of the four dimensions of social capital above (ISC, FSC, CSC, and SSC) were adapted from the scales of a validated Chinese version of Health-related Social Capital Measurement (20). According to characteristics of the living and studying environments of the youth, we tailored the 15 items in four dimensions (Table 1). The answer to each item ranges from 1 (strongly disagree) to 5 (strongly agree), with a higher total score indicating stronger social capital.

**Table 1:** The percentages of the participating Chinese youths who (strongly) agreed each survey question of social capital before and after the COVID-19 lockdown in the COVID-19 Impact on Lifestyle Change Survey (COINLICS).

**Variables**		**All (*n =* 10,540)**	**High school (*n =* 2,855)**	**Undergraduate (*n =* 7,419)**	**Graduate (*n =* 266)**
**Individual social capital**					
Q1	You have many close contacts.				
	Before lockdown	7.3	4.3	8.2	11.7
	After lockdown	4.0	2.8	4.5	4.5
Q2	You have many social interactions with people other than your family members.				
	Before lockdown	8.6	5.5	9.4	16.9
	After lockdown	5.9	4.1	6.6	6.8
Q3	You always trust people who have social interaction with you.				
	Before lockdown	60.4	49.9	64.1	70.3
	After lockdown	58.5	47.4	62.5	67.3
Q4	You always receive emotional/financial/instrumental support from friends/classmates.				
	Before lockdown	54.2	43.5	57.8	66.5
	After lockdown	54.2	43.0	58.0	65.8
Q5	You have a good relationship with your classmates.				
	Before lockdown	71.8	61.4	75.5	80.5
	After lockdown	70.4	59.7	74.2	79.7
**Family social capital**					
Q6	You live with family members.				
	Before lockdown	74.1	87.6	70.1	38.7
	After lockdown	90.2	93.1	89.5	80.1
Q7	You have a good relationship with your family (mainly including parents, brothers and sisters).				
	Before lockdown	81.6	75.9	83.5	87.6
	After lockdown	79.2	74.5	80.8	85.0
Q8	You always receive emotional/financial/instrumental support from family members.				
	Before lockdown	73.5	64.2	76.9	79.0
	After lockdown	73.7	64.3	77.1	80.5
**Community social capital**					
Q9	You frequently participate in activities organized by community organizations.				
	Before lockdown	13.8	4.7	17.5	9.0
	After lockdown	10.9	3.5	13.9	7.9
Q10	You always receive support from community organizations.				
	Before lockdown	8.4	4.7	9.9	6.4
	After lockdown	7.9	4.4	9.2	7.1
Q11	You always receive emotional/financial/instrumental support from your teachers or instructors.				
	Before lockdown	26.0	20.9	27.8	30.8
	After lockdown	29.3	22.2	31.8	35.7
Q12	You are very concerned about what happens in the same community/dormitory building.				
	Before lockdown	36.5	33.7	37.3	45.1
	After lockdown	41.4	35.8	43.0	54.9
Q13	You agree that people who live in the same community/dormitory can be trusted.				
	Before lockdown	24.2	25.7	31.6	25.4
	After lockdown	24.6	26.4	33.1	26.1
**Society social capital**					
Q14	You trust other health organizations/governmental organizations very much.				
	Before lockdown	57.7	64.7	55.1	56.8
	After lockdown	60.6	66.6	58.3	59.4
Q15	You agree with the statement that talented people will be recognized by the society.				
	Before lockdown	46.9	46.4	46.8	54.5
	After lockdown	48.0	47.4	48.1	51.9

### Statistical Analysis

Descriptive statistics were used for the participants' demographic characteristics and social capital, with mean and standard deviation (SD) for continuous variables, and percentages for categorical variables. Differences in demographic characteristics, the changes of social capital before and after the lockdown, and the frequency differences at the individual level among youths of different educational levels were compared based on *t*-tests/ANOVA for continuous variables, or χ^2^ tests for categorical variables. R 3.6.2 was used to perform all statistical analyses. Statistical significance was declared if a two-sided *p* < 0.05.

## Results

Of 10,540 participants in the study, 2,855 participants were high school students, 7,419 participants were undergraduate students, and 266 participants were graduate school students (Table 2). The participants aged from 15 to 33 years, with a mean age of 19.9±2.3. Most of them were female (71.3%), of Han ethnicity (94.9%), non-urban residents (61.8%), and from the west region (87.1%). Around half of the participants had a household income of 12,000–60,000 *yuan* per year. Significant differences were observed for all demographic characteristics among the three educational levels. More specifically, the percentages of urban residents were higher in undergraduate students (42.6%) and graduate students (62.8%) than in high school students (24.3%), and no high school students from the central region were enrolled.

**Table 2:** Baseline characteristics of the participating youths in the COVID-19 Impact on Lifestyle Change Survey (COINLICS).

**Variable**	**Mean (SD) or percentage (%)**				
	**All (*n =* 10,540)**	**High school (*n =* 2,855)**	**Undergraduate (*n =* 7,419)**	**Graduate (*n =* 266)**	***P*-value**
**Age** (year)	19.9 (2.3)	17.5 (1.2)	20.6 (1.8)	24.7 (3.4)	** <0.001**
**Sex**					** <0.001**
Male	28.7	24.2	30.4	27.8	
Female	71.3	75.8	69.6	72.2	
**Ethnicity**					** <0.001**
Han	94.9	96.7	94.4	91.0	
Minority	5.1	3.3	5.6	9.0	
**Urbanicity**					** <0.001**
Urban	38.2	24.3	42.6	62.8	
Non-urban	61.8	75.7	57.4	37.2	
**Region** ^a^					** <0.001**
Northeast	0.3	0.1	0.3	3.4	
East	9.2	0.7	11.9	25.2	
West	87.1	99.2	83.5	56.8	
Central	3.4	0.0	4.3	14.6	
**Household income (** ***yuan*** **/year)**					** <0.001**
<12,000	20.0	24.5	18.7	6.8	
≥12,000–20,000	28.0	35.6	25.8	8.3	
>20,000–60,000	26.7	25.8	27.3	21.8	
>60,000–10,0000	13.1	9.5	14.1	23.3	
>100,000–200,000	8.6	3.3	10.0	25.9	
>200,000	3.6	1.3	4.1	13.9	
**Major**					** <0.001**
Medical Science	37.0	88.7	16.5	52.3	
Science/Engineering	25.8	10.5	31.7	26.3	
Social Science	37.2	0.8	51.8	21.4	

a *Northeast (Liaoning, Jilin, Heilongjiang province), East (Beijing, Tianjin, Hebei, Shanghai, Jiangsu, Zhejiang, Fujian, Shandong, Guangdong, Hainan province), Central (Shanxi, Anhui, Jiangxi, Henan, Hubei, Hunan province), and West of China (Inner Mongolia, Guangxi, Chongqing, Sichuan, Guizhou, Yunnan, Tibet, Shaanxi, Gansu, Qinghai, Ningxia, Xinjiang). All significant p-values (p < 0.05) were bolded*.

The score of all dimensions of social capital showed significant differences (all *p* < 0.001) among three educational levels both before and after the lockdown (Table 3). Overall, the ISC score decreased from 15.1 to 14.8 and CSC score decreased from 13.4 to 13.1, while the FSC score increased from 12.7 to 13.0 and SSC score increased from 7.1 to 7.2 (all *p* < 0.001). In different educational groups, the ISC score and CSC decreased in all subgroups (all *p* < 0.01); the FSC score of undergraduate students increased (*p* < 0.001); and the SSC score increased in all subgroups (all *p* < 0.05).

**Table 3:** Changes in social capital before and after the COVID-19 lockdown among the participating youths in the COVID-19 Impact on Lifestyle Change Survey (COINLICS).

**Variable**	**Median [p25, p75] or percentage (%)**				***P*-value^**†**^**
	**All (*n =* 10,540)**	**High school (*n =* 2,855)**	**Undergraduate (*n =* 7,419)**	**Graduate (*n =* 266)**	
**Population-level**					
**Individual social capital**					
Before lockdown	15 (13, 17)	14 (12, 16)	16 (13, 17)	14 (12, 16)	** <0.001**
After lockdown	15 (13, 17)***	14 (12, 16)***	15 (13, 17)***	14 (12, 16)***	** <0.001**
**Family social capital**					
Before lockdown	13 (11, 15)	13 (11, 15)	13 (11, 15)	13 (11, 15)	** <0.001**
After lockdown	13 (12, 15)***	13 (11, 15)	14 (12, 15)***	13 (11, 15)	** <0.001**
**Community social capital**					
Before lockdown	13 (11, 15)	12 (10, 14)	14 (11, 16)	12 (10, 14)	** <0.001**
After lockdown	13 (11, 15)***	12 (10, 14)**	13 (11, 15)***	12 (10, 14)**	** <0.001**
**Society social capital**					
Before lockdown	7 (6, 8)	7 (6, 8)	7 (6, 8)	7 (6, 8)	** <0.001**
After lockdown	7 (6, 8)***	7 (6, 8)*	7 (6, 8)***	7 (6, 8)*	** <0.001**
**Individual-level**					
Individual social capital					** <0.001**
Increased	17.0	17.3	16.9	17.3	
Constant	52.5	54.9	52.1	36.8	
Decreased	30.5	27.7	31.0	45.9	
Family social capital					** <0.001**
Increased	21.7	11.7	24.7	47.0	
Constant	69.1	80.0	65.8	43.2	
Decreased	9.2	8.3	9.5	9.8	
Community social capital					** <0.001**
Increased	19.1	13.6	21.1	22.9	
Constant	52.6	63.1	48.7	47.4	
Decreased	28.4	23.3	30.3	29.7	
Society social capital					**0.025**
Increased	10.3	9.7	10.6	7.5	
Constant	85.8	86.8	85.4	85.7	
Decreased	3.9	3.5	4.0	6.8	

Values under a given variable were marked by asterisks, if the difference before and after COVID-19 lockdown within a given educational level was significant (*p < 0.05, **p < 0.01, ***p < 0.001).

† *P-values tested the significance of the differences in each variable across educational levels. All P-values were based on t-tests/ANOVA for continuous variables or χ^2^ tests for categorical variables. All significant p-values (p < 0.05) were bolded*.

At the individual level, most of the youths participating in the study showed constant social capital scores between the two time points, with the percentage ranging from 52.5 to 85.8% across four scales (Table 3). In addition, overall, more participants had decreased than increased ISC scores (30.5% vs. 17.0%) and CSC scores (28.4% vs. 19.1%), and more participants had increased rather than decreased FSC scores (21.7% vs. 9.2%) and SSC scores (10.3% vs. 3.9%). Participants at different educational levels also showed the same trend as the whole group. Among graduate students, 45.9% of participants had decreased ISC score and 47.0% of them had increased FSC score, which were higher than the other groups; among undergraduate students, the percentages of the participants with decreased CSC (30.3%) and increased SSC (10.6%) scores were higher than the other groups. Differences in the composition ratio of individual-level changes among educational levels were found (all *p* < 0.05) in all dimensions of social capital.

## Discussion

This is a retrospective study based on a national sample, which provided a picture of changed social capital among youths before and after the lockdown. We found significant changes in social capital of all dimensions across educational levels, except for the FSC in high school and graduate students. At the individual level, most youths' social capital after lockdown was constant compared to before lockdown. However, more youths showed a decline in their ISC and CSC than those showed an ascent; more youths showed an ascent in their FSC and SSC than those with decreased scores. Heterogeneities in social capital changes existed across educational levels.

Several explanations may account for the changes in social capital among youths, especially regarding the significant decline of ISC and CSC. Previous research has suggested that social contact and community participation among population might be disrupted in the face of a disaster or catastrophe (e.g., earthquake or tsunami) (7). During the COVID-19 outbreak, although the lockdown in China was lifted in April, social distancing was still recommended, and parents may adopt the advice to prevent youths away from networking activities (e.g., wedding, club parties, classmate gathering). These measures may affect their social contact especially with their friends and community, as online communication platforms cannot compensate for the emotional demands of face-to-face communication and community participation (21). In addition, the decline might also attribute to the adverse mental health status affected by COVID-19, which was inconducive to interpersonal communication.

Different from previous studies on post-disaster social capital concerns (7), the improvements in FSC and SSC found in this study suggested that the impact of COVID-19 on social capital is not entirely negative. COVID-19 and the accompanying lockdown, in some sense, granted opportunities for family members to communicate internally, which might account for the improvement in FSC. In terms of SSC, the possible mechanism accounted for the increase might be the government's effective disaster management and social governance. Specifically, the Chinese public has a high level of trust in the government. In face of the disaster, the Chinese government and health agencies has actively and rationally taken countermeasures during the epidemic to curb the spread of COVID-19, which strengthened the social cohesion. All industries (especially the health industry) were united against COVID-19, and positive news reports promoted solidarity. Furthermore, the lockdown lifted *per se* demonstrated the effectiveness of national unity in the fight against the pandemic, which may have profoundly strengthened the social capital among youths. These factors might grant youths the spirit of solidarity and sense of security in facing the disaster, thus increasing the SSC to some extent (22).

There are some suggestions to policy-makers and health professionals on the basis of our findings. For example, to prevent further decreases in or even increase ISC and CSC among youths, health professionals could collaborate with schools to develop online peer communication activities and thus provide emotional support (14); policy-makers should take measures to improve community services, and develop guidelines and instructions to anticipate the needs of vulnerable youths, especially those who used to take less advantage of social capital (23). To maintain or further increase SSC, relevant authorities may strengthen the monitoring of social media to curb the spread of false information. In addition, what aroused our concern is that the decline in ISC and CSC may persist even after lockdown. Since a previous study suggested that the coronavirus may have a long-term transmission trend (24), social distancing is still inevitable. New strategies are needed to reshape social capital especially the ISC and CSC. For example, opening some public places under strict monitoring in low-risk areas (such as cinemas and bookstores) may promote the participation of community activities. Since many countries are still under lockdown, we hope our study could provide some references for other countries or regions. Our study also found the heterogeneities in social capital changes existed across educational levels before and after the lockdown, which implies that policy-makers should take into consideration the educational level and types of social capital while developing tailored interventions for recovery of social capital.

Our study has some limitations. First, since our study measures a before and after scenario by asking about the “before” retrospectively and the social capital data are self-reported, there may be recall and reporting bias; particularly, this recall was made during a traumatic ongoing event, which may further skew the perception of all levels of social capital (e.g., being under-perceived) due to negative or depressive emotion during the long-lasting pandemic. However, the self-assessment of social capital at two time points might reflect their perceived changes which are usually closely correlated with their actual changes (16, 25, 26). Second, we only measured two time points in this study, thus were not able to track the dynamic trends of social capital during the whole period (27). Third, this study was conducted based on a national convenience sample that may not be fully representative of the Chinese youth. Using a snowball sampling technique may lead to some notable skewness in the collected data (28, 29), such as a considerably large proportion of females and youths from western regions of China in our study. Besides, all participating youths were students, so the results may not be extrapolated to other youth groups (e.g., out-of-school youth). However, this large convenience sample, promptly recruited online, presents unique strengths by drawing important conclusions from the targeted population during the epidemic without risk of infection. Note that this approach and the resultant findings may differ in the context of many natural disasters (e.g., earthquake, tsunami) which can cause the loss of ability to stay in touch or trade information via electronic means, and thus affect the ability to build or use social capital.

Our large-scale nationwide study suggested the changes of social capital among the Chinese youths before and after the COVID-19 lockdown. Specifically, the social capital at individual and community level generally declined, while the family-level social capital and society-level social capital generally ascended. Our findings would inform policy-makers and health professionals of the changed social capital among youths during COVID-19 lockdown, for better policy making and clinical practice to improve youths' mental health in the post-COVID era. School administrators should also be informed of these changes, so in-class and extracurricular programs could be designed to counteract them. Although our findings also serve as important references for other countries or regions in which lockdown measures are in effect or to be (re)considered, perceptions on and changes in social capital, especially SSC, under similar situations in those countries and regions with more individualistic subcultures and/or less trust in governments may be significantly different or even reversed. Therefore, more efforts in the countries of different cultures are warranted to increase all dimensions of social capital in adaptive approaches.

## Data Availability Statement

The raw data supporting the conclusions of this article will be made available by the authors, without undue reservation.

## Ethics Statement

The studies involving human participants were reviewed and approved by the Sichuan University Medical Ethical Review Board (KS2020414). Written informed consent to participate in this study was provided by the participants' legal guardian/next of kin.

## Author Contributions

BY, MLu, SY, and PJ took a principal role in designing the study, writing the protocol, developing methodologies, analyzing the data, and drafting the manuscript. PJ contributed to the proposal writing and made a critical revision to the manuscript. JZ contributed to the write up of the study protocol and made a revision to the manuscript. MLi and BY performed the statistical analysis. SY and PJ supervised the study and edited the manuscript. All authors read and approved the final manuscript.

## Conflict of Interest

The authors declare that the research was conducted in the absence of any commercial or financial relationships that could be construed as a potential conflict of interest.

## Publisher's Note

All claims expressed in this article are solely those of the authors and do not necessarily represent those of their affiliated organizations, or those of the publisher, the editors and the reviewers. Any product that may be evaluated in this article, or claim that may be made by its manufacturer, is not guaranteed or endorsed by the publisher.
